# Habitat Degradation Facilitates the Invasion of Neophytes: A Resurvey Study Based on Permanent Vegetation Plots in Oak Forests in Slovenia (Europe)

**DOI:** 10.3390/plants13070962

**Published:** 2024-03-27

**Authors:** Janez Kermavnar, Lado Kutnar

**Affiliations:** Department of Forest Ecology, Slovenian Forestry Institute, Večna pot 2, 1000 Ljubljana, Slovenia; lado.kutnar@gozdis.si

**Keywords:** long-term vegetation change, invasive non-native plants, oak mortality, *Quercus robur*, *Quercus petraea*, canopy openness, *Impatiens parviflora*, Slovenia

## Abstract

The spread of neophytes (non-native plant species) challenges the conservation status and ecological integrity of forests, especially in lowland areas. Long-term resurvey studies are needed to evaluate the temporal dynamics of neophytes in forests; however, such data are scarce. In 2023, we resampled a set of 45 permanent vegetation plots (established in 1992/93) in two forest vegetation types: oak–hornbeam forests dominated by *Quercus robur* and colline oak–beech forests dominated by *Q. petraea*. Over the last 30 years, oak forests have experienced extensive oak tree mortality, with the degree of habitat degradation being greater in *Q. robur* forests. In the early 1990s, only three neophytes with low abundance were recorded across all plots. In the 2023 resurvey, the total number of neophytes increased to 22 species (15 herbaceous and 7 woody species), comprising 6.9% of the total species pool in the understory layer. The increase in the plot-level number and cover of neophytes was significant in plots dominated by *Q. robur* but not in those with *Q. petraea*. The most frequent neophytes were *Impatiens parviflora* (present in 31% of plots), *Solidago gigantea* (27%), *Erigeron annuus* (16%) and *Erechtites hieraciifolia* (16%). The richness and cover of neophytes were significantly affected by the tree layer cover (negative correlation) and the degree of soil disturbance (positive correlation). All neophytes established in disturbed patches, whereas the occurrence of *I. parviflora* was exceptional as it was able to colonize less degraded, shaded understory environments. Habitat degradation (the mortality-induced loss of stand-forming oak trees resulting in extensive tree layer cover decrease) emerged as a key driver promoting neophyte proliferation, coupled with the impact of management-induced disturbances affecting overstory and soil conditions. The spread is expected to continue or even intensify in the future because novel light regimes and disturbances make forest habitats less resistant to neophyte proliferation.

## 1. Introduction

Land-use change, habitat loss and fragmentation, climate change and biological invasions have been identified as significant contributors to the accelerating decline in biodiversity worldwide [1,2]. Habitat degradation refers to a decrease in the quality of a habitat’s condition. Forest habitat degradation is generally understood as the cumulative impact of human-originated processes that directly and indirectly affect the naturalness and conservation status of forest habitat in a negative way, with the potential to cause irreversible changes to its long-term persistence [3].

Many forest vegetation types, such as oak-dominated forests located in lowlands near human settlements, have been under pressure from various anthropogenic disturbances for centuries [4,5]. These impacts are intensifying due to an increasing number of local (e.g., tree mortality) and global (e.g., climate warming) threats. The increase in trade activity, intentional introductions and the use of non-native ornamental plants in horticulture, alongside anthropogenic habitat disturbances, rising temperatures and soil eutrophication (nitrogen deposition, intensive agriculture), facilitate the establishment and spread of non-native plant species, i.e., neophytes [6,7]. The invasive spread of neophytes is causing significant alterations of forest ecosystems on multiple levels [8,9,10,11]. Their proliferation can be seen as a co-occurring process driven by habitat degradation and represents one of the greatest challenges to the biotic diversity of forest communities [12], causing its decline and homogenization.

Invasion ecology has emphasized the central role of forest disturbance in facilitating neophyte proliferation [6,13,14]. By increasing resource availability for plant growth (e.g., light, nutrients, water) and altering competitive interactions within resident vegetation, disturbances create favorable niches for disturbance-dependent taxa to enter the plant community, particularly if these changes coincide with the supply of suitable propagules [15,16]. Altered ecological conditions coupled with the greater availability of resources in the initial stages after disturbance favor neophytes that exploit disturbed forest habitats for their spread [10,17,18]. A denser forest canopy and more natural site conditions are negatively correlated with plant invasions, while intensive management, nutrient enrichment and the human-assisted migration of plant diaspores (anthropochory) increase the susceptibility of forests to the spread of neophytes [13,19]. It has been proposed that certain neophytes are better adapted to disturbances, making them more effective dispersers and competitors under such conditions than native plant species [20]. The majority of invasive species exhibit early successional traits (e.g., shade intolerance, fast growth, great ability for reproduction and dispersion), but certain woody and herbaceous neophytes are also able to thrive in deeply shaded forests [14].

Over the past few decades, lowland *Quercus robur* L. forests in Europe have experienced significant crown defoliation, tree dieback and consequent canopy cover decline [4,21,22]. Since the 1990s, oak mortality has been considered to be a complex process caused by a combination of abiotic and biotic factors, with anthropogenic interventions in the hydrological regime (the lowering of the groundwater table) playing an important role [23]. The extensive loss of canopy cover driven by the mortality of dominant tree species has the potential to change the structure and diversity of forest vegetation [24].

The comparison of resurveyed vegetation data with original surveys performed several decades ago provides insights into the temporal trends of understory composition. Vegetation resurvey data are increasingly used to evaluate plant community responses to global environmental change [25,26]. In this context, permanent vegetation plots with known locations are especially valuable. The resurvey of historical vegetation plots can reveal mechanisms underlaying plant invasions. However, the spread of neophytes can result from various ecological changes [5,27], depending primarily on the characteristics of the invading species [28]. While their presence was found to be promoted by proximity to settlements, high temperatures at lower elevations and sparse or damaged forest areas, some studies have observed an increasing trend of neophyte occurrences in relatively undisturbed lowland forests [5], including unmanaged forest reserves [29]. In general, riparian and floodplain forests are frequently reported for their high share of neophytes and their increasing trend over time [27,28,30].

**Figure 1:**
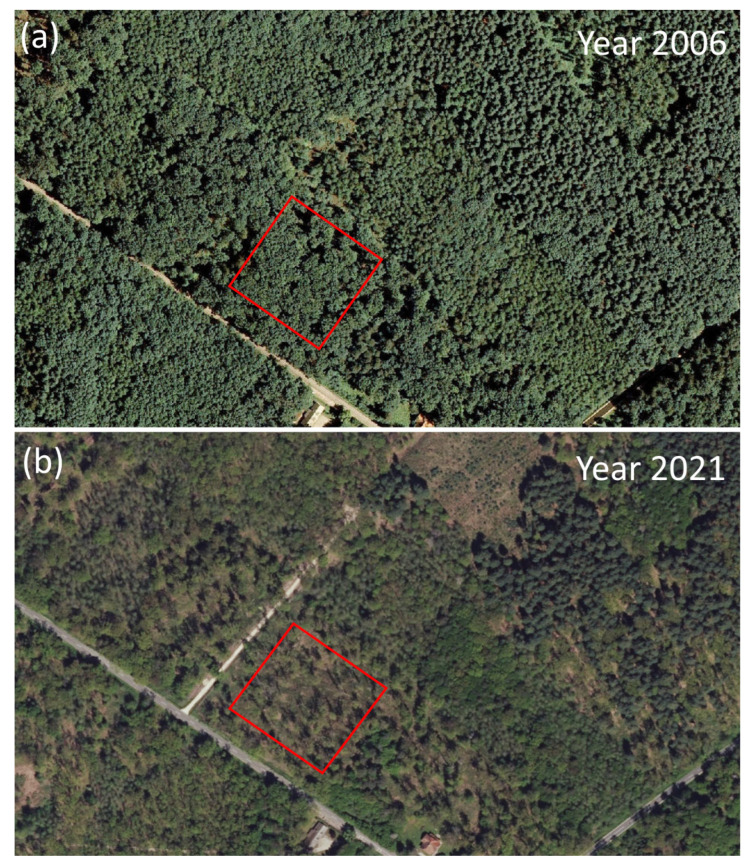
Satellite images from (**a**) 2006 and (**b**) 2021 for the “Dobrava” study site in a lowland oak–hornbeam forest dominated by *Quercus robur*, Slovenia. The red rectangle denotes a 100 × 100 m permanent research area established in 1993. The comparison reveals significant changes in stand conditions on the research plot and overall habitat degradation on the landscape level (i.e., more open and fragmented forests). Source: Slovenian Environment Agency—ARSO [31].

Using consistent sampling methodology across selected Slovenian sites, we revisited permanent vegetation plots with precise geolocation 30 years after their establishment. We aimed at quantifying the 1992/93-2023 changes in the occurrence, number and cover of neophytes in two forest vegetation types: (i) lowland oak–hornbeam forests dominated by *Quercus robur* and (ii) colline oak–beech forests dominated by *Q. petraea* (Matt.) Liebl. In response to changes in the overstory layer induced by oak mortality, forest management disturbances and overall habitat degradation (Figure 1), an increase in the number and abundance of neophytes was hypothesized. The aims of our study were (i) to compare historical and recent vegetation surveys to determine if there is a significant increase in the number and cover of neophytes over time, (ii) to compare the occurrence patterns of neophytes in two different oak-dominated forest types and (iii) to explain the relationship between ecological factors and the level of invasion.

## 2. Results

### 2.1. Patterns of Neophyte Occurrence

**Figure 2:**
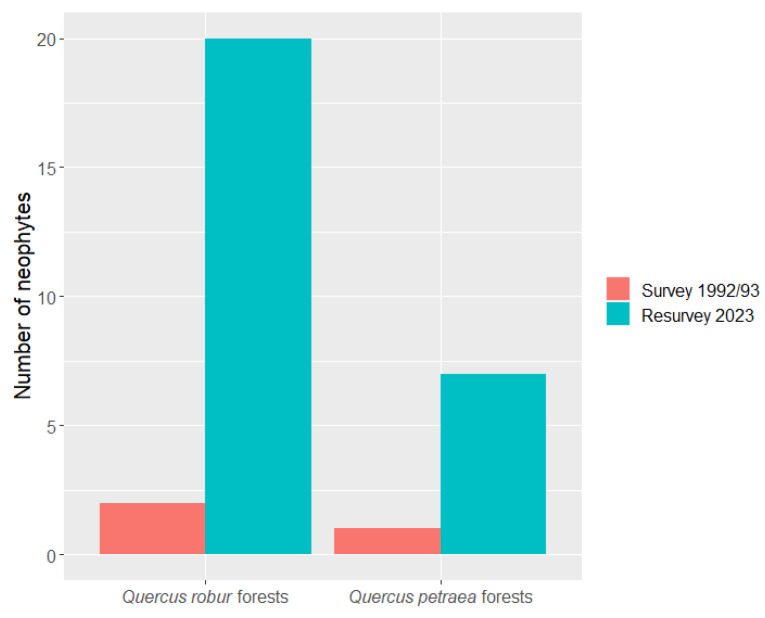
The total number of neophytes recorded on 25 plots in lowland oak–hornbeam forests dominated by *Quercus robur* and on 20 plots in colline oak–beech forests dominated by *Q. petraea*. The original survey was conducted in 1992/93, while the resurvey took place in 2023.

In lowland oak–hornbeam forests dominated by *Quercus robur*, two neophytes with low abundance were recorded during the 1992/93 survey. *Veronica persica* Poir. was present on three plots, and one plot was occupied by saplings of the non-native tree *Pinus strobus* L. In the 2023 resurvey, however, the total number of neophytes increased to 20 plant species (14 herbaceous and 6 woody species; Figure 2). The most frequent were *Impatiens parviflora* DC. (present on 14 out of 25 plots) and *Solidago gigantea* Aiton (12 plots), followed by *Erechtites hieraciifolia* (L.) Rafin. ex DC. (7) and *Erigeron annuus* (L.) Pers. (6). *Impatiens parviflora*, *Solidago gigantea* and *Erigeron annuus* had a mean cover higher than 5% (Table 1).

In colline oak–beech forests dominated by *Quercus petraea*, one neophyte (*Conyza canadensis* (L.) Cronq.) was recorded in the 1992/93 survey. In the 2023 resurvey, the total number of neophytes increased to seven species (five herbaceous and two woody species; Figure 2). All neophytes were present with minimal cover (Table 2).

### 2.2. Changes in Richness and Cover of Neophytes

**Figure 3:**
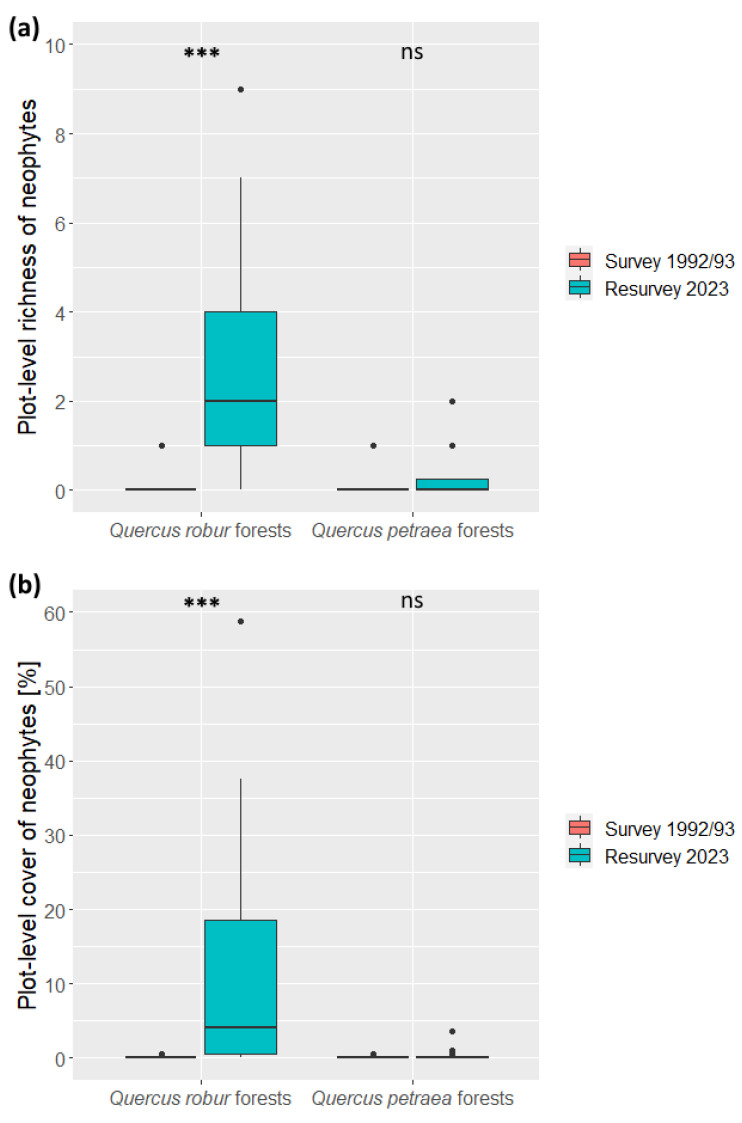
Temporal changes in the (**a**) plot-level richness and (**b**) plot-level cover of neophytes recorded in two oak-dominated forest vegetation types: lowland oak–hornbeam (*Quercus robur*) forests and colline oak–beech (*Quercus petraea*) forests. The original survey was conducted in 1992/93, while the resurvey took place in 2023. The difference between the two sampling periods was tested with the Wilcoxon test: *** *p* < 0.001, ns—not significant changes.

In the *Quercus robur*-dominated forests, the mean plot-level richness increased significantly (Wilcoxon test: *p* < 0.001) from 0.16 in the 1992/93 survey to 2.76 in the 2023 resurvey (Figure 3a). In the resurvey, the highest richness of neophytes was nine per plot (i.e., 22.5% of the total understory richness), and 19 plots (out of 25) contained at least one neophyte. The mean plot-level cover of neophytes also increased significantly (Wilcoxon test: *p* < 0.001), from 0.09% in the 1992/93 survey to 10.28% in the 2023 resurvey. In the resurvey, the highest neophyte cover reached almost 60% of the sampling plot area (Figure 3b).

In contrast, the *Quercus petraea*-dominated forests did not exhibit a significant increase in the plot-level richness and cover of neophytes (Figure 3a,b). Out of 20 resurveyed plots, 15 (75%) did not contain any neophyte species. In relative terms, the maximum share of neophytes in the total understory richness was 6.1%, and their maximum plot-level cover represented only 1.2% of the total understory cover.

### 2.3. Neophytes in Relation to Tree Layer Cover and Soil Disturbance

**Figure 4:**
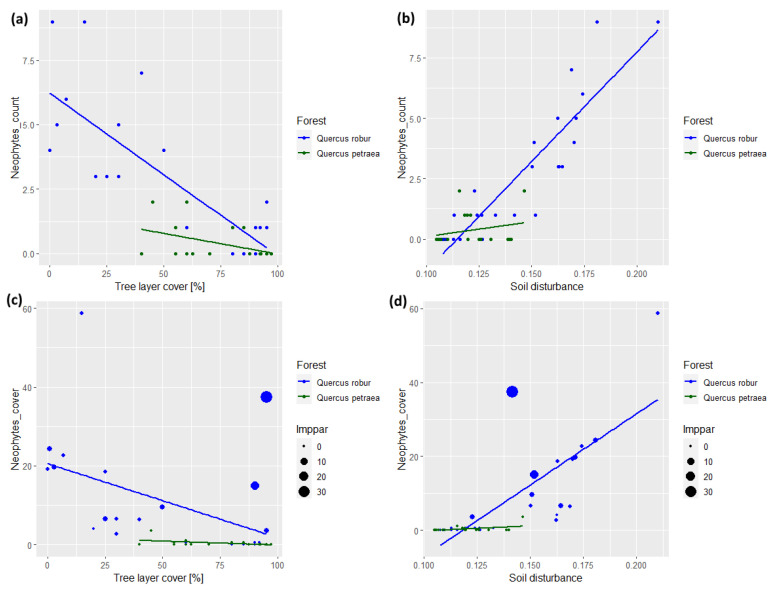
Linear models for the (**a**,**b**) plot-level richness (species count) and (**c**,**d**) cover (%) of neophytes along tree layer cover (**a**,**c**) and soil disturbance gradients (**b**,**d**), based on data from the 2023 vegetation resurvey. The soil disturbance gradient was calculated as the community-weighted mean of species’ disturbance indicator values, which ranged from 0.105 to 0.210 across all plots. Datapoints and regression lines are shown for lowland oak–hornbeam forests dominated by *Quercus robur* (blue) and for colline oak–beech forests dominated by *Q. petraea* (green). Note the difference in the range of the tree layer cover and soil disturbance between the two forest types. For panels c and d, different sizes of dots for *Q. robur* forests depict the gradient of abundance for *Impatiens parviflora* (Imppar), the most frequent neophyte in our dataset.

In the *Quercus robur*-dominated forests, the plot-level richness of neophytes showed a significant negative relationship with tree layer cover (*p* < 0.001, pseudo-R^2^ = 0.643; Figure 4a). The plot-level cover of neophytes was also negatively correlated with tree layer cover (Figure 4c). However, this relationship was much weaker (*p* < 0.05; pseudo-R^2^ = 0.189) because some closed canopy plots exhibited substantial neophyte cover. This pattern was exclusively driven by *Impatiens parviflora* (Figure 4c). The plot-level richness of neophytes in the *Q. robur*-dominated forests was strongly positively correlated with the soil disturbance gradient (*p* < 0.001, pseudo-R^2^ = 0.726; Figure 4b). A significant positive correlation was detected for plot-level neophyte cover as well (*p* < 0.001, pseudo-R^2^ = 0.535; Figure 4d).

In the *Quercus petraea*-dominated forests, the response of neophytes to two explanatory variables was evidently weaker. Nevertheless, we found a significant negative relationship between the plot-level richness of neophytes and tree layer cover (*p* < 0.05, pseudo-R^2^ = 0.224; Figure 4a). The plot-level cover of neophytes also decreased along the gradient of tree layer cover (*p* < 0.05, pseudo-R^2^ = 0.175; Figure 4c). In contrast, the richness and cover of neophytes in the *Quercus petraea*-dominated forests did not respond significantly to the soil disturbance gradient (Figure 4b,d).

### 2.4. Species Response Curves

**Figure 5:**
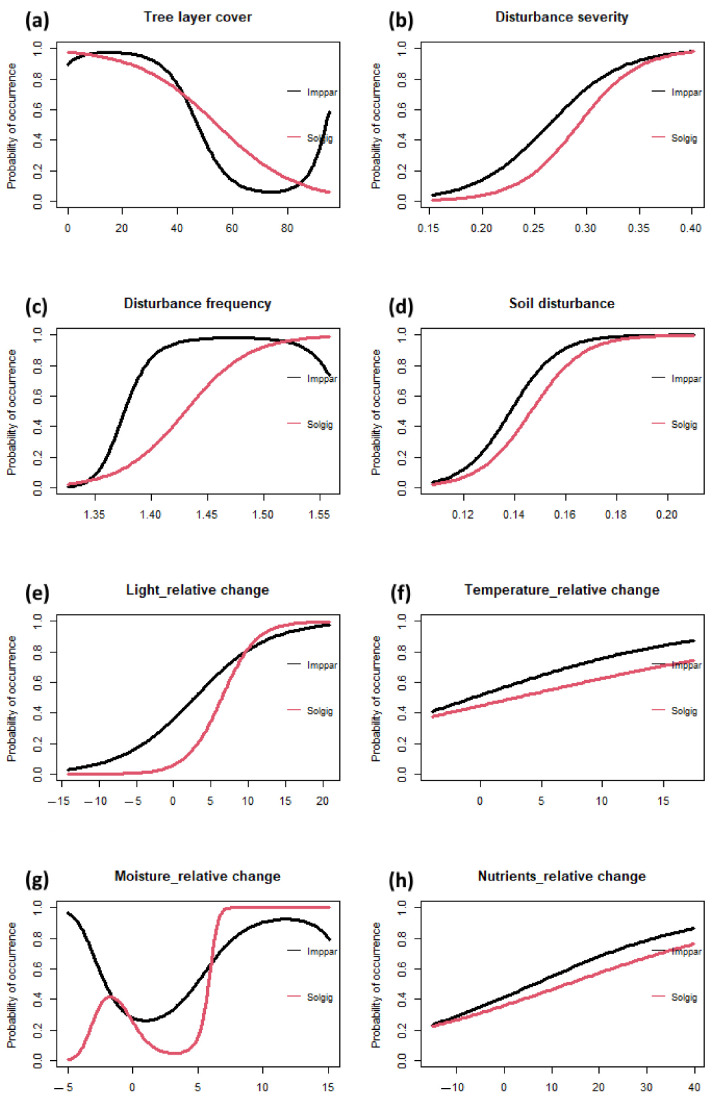
Species response curves for *Impatiens parviflora* (Imppar, black lines; present on 14 plots) and *Solidago gigantea* (Solgig, red lines; present on 12 plots) along gradients of selected ecological predictors: (**a**) tree layer cover, (**b**) disturbance severity, (**c**) disturbance frequency, (**d**) soil disturbance and the relative changes in (**e**) light conditions, (**f**) temperature, (**g**) soil moisture and (**h**) soil nutrients. The analysis was based on data for resurveyed plots in lowland oak–hornbeam forests dominated by *Quercus robur*. For details on model statistics, see Table 3.

We present the response curves of *Impatiens parviflora* and *Solidago gigantea*, the most frequent neophytes on plots in *Quercus robur*-dominated forests (Figure 5). These two species exhibited a strong and similar response to the majority of ecological predictors, such as disturbance severity (Figure 5b), soil disturbance (Figure 5d) and relative changes in temperature (Figure 5f) and nutrients (Figure 5h). However, for some other ecological factors, clear differences in their response can be observed. For example, *S. gigantea* strongly decreased along the tree layer cover gradient, whereas *I. parviflora* was also abundant on some plots in closed stands with high tree layer cover (Figure 5a). Generally, their probability of occurrence was enhanced by higher light levels, higher temperatures and greater nutrient availability (although for temperature and nutrients, the percentage of explained deviance was low; Table 3). The most complex response curves were observed for the relative change in moisture. Both species showed a preference for plots where soil moisture increased but were also present on plots with a negative change in moisture (Figure 5g). This pattern was more pronounced for *I. parviflora* than for *S. gigantea*, suggesting a wider ecological niche for *I. parviflora* in terms of soil moisture.

## 3. Discussion

### 3.1. Oak Mortality Promotes the Spread of Neophytes

We investigated the effect of habitat degradation in the form of tree mortality and natural or management-related disturbances on the long-term temporal pattern of neophyte occurrence. Our resurvey study revealed that the total number, plot-level species richness and cover of neophytes have increased in oak forests in Slovenia over the last 30 years. Neophytes, which were very rare in the early 1990s, have become more frequent and abundant in the resurveyed plots. However, the increase was significant in lowland oak–hornbeam forests dominated by *Quercus robur* but not in colline oak–beech forests dominated by *Q. petraea*. This difference can be attributed to the fact that mortality and the overall disturbance intensity were much higher in lowland oak–hornbeam forests dominated by *Q. robur* [4,23].

**Figure 6:**
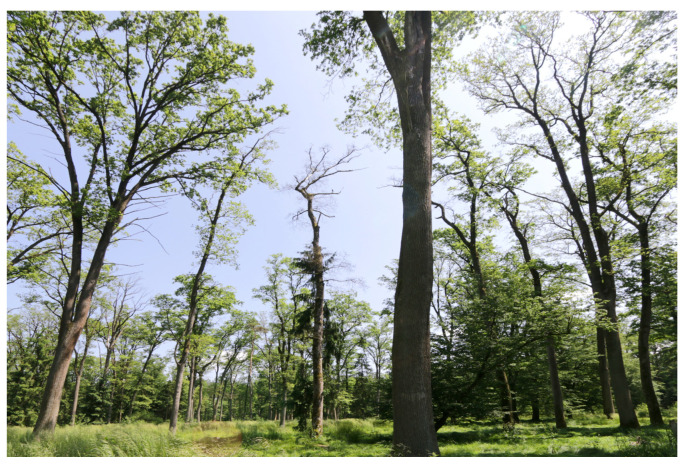
The massive crown defoliation and mortality of mature oak trees (*Quercus robur*) in the 2023 resurvey of permanent vegetation plots at the “Cigonca” study site (lowland oak–hornbeam forest). Photo: Lado Kutnar.

Mature oak trees exhibited profound crown defoliation or complete dieback, altering the ecological conditions at the forest floor (Figure 6). Across the resurveyed plots, tree layer canopy cover varied from closed stands to nearly treeless disturbed patches. Disturbances caused the opening of tree canopies and contributed to the formation of empty niches, which in turn promoted the observed neophyte proliferation by increasing the availability of light and other resources [32]. Elevated solar radiation in canopy gaps likely boosted the litter decomposition rate and mineralization, leading to faster nutrient turnover and greater nutrient supply for plant growth [33]. Typically, fast-growing and quick-to-colonize competitive and ruderal plants opportunistically capitalize on such resource pulses. Disturbed, less dense forests are far more susceptible to neophyte invasion compared to preserved, closed canopy stands. This confirms the general notation that non-native plant species benefit from disturbance [34] and that neophytes are more prolific predominantly on degraded sites where the tree canopy has been disrupted [35].

We found a clear relationship between disturbance intensity and the number and cover of neophytes, in line with previous findings [18,36]. In both forest types, the number and abundance of neophytes were negatively associated with tree layer canopy cover. The most intensely disturbed plots, with tree layer cover below 20%, shifted towards an early successional stage dominated by competitive ruderal species, including neophytes. In general, preserved forests are considered more resistant to neophyte proliferation due to limited light availability in the understory [16,19]. However, disturbance and abiotic stress may open tree canopies and promote invasion [32]. Changes in environmental conditions and the high availability of resources can be a significant trigger for the proliferation of neophytes in forest ecosystems. Forest disturbances facilitate the spread of synanthropic species with highly competitive strategies, which tend to quickly dominate in terms of biomass and ultimately eliminate less competitive, rare native species [37].

We identified 22 different neophyte species across 45 resurveyed permanent vegetation plots. For comparison, Marinšek and Kutnar [9] identified 15 neophytes across a much higher number of sampling plots along the Mura River (Slovenia) and concluded that oak–hornbeam forests were less invaded compared to alluvial forests with *Alnus glutinosa* L. Gaertn. and *Fraxinus excelsior* L. in the overstory. They also found a strong negative correlation between neophyte abundance and tree layer cover, similar to our main conclusion.

In addition to oak mortality and the reduction in tree layer cover, the difference in neophyte invasion between lowland *Q. robur* and colline *Q. petraea* forests may be due to the landscape context itself. *Q. robur* forests in the lowlands depend on the natural hydrological regime (occasional flooding, high water table), now disrupted by intense human activities. The depletion of groundwater levels, drainage of forest sites and soil drying are expected to worsen under climatic stress (summer heatwaves and droughts). Lowland oak–hornbeam forests are surrounded by intensively cultivated fields, settlements or urbanized areas and disturbed habitats, whereas *Q. petraea* forests are located more in the colline belt, further away from direct anthropogenic influences. Over the last three decades, lowland landscapes have likely experienced an increase in the proportion of intensified agricultural areas and other non-forest land uses, which increases the deposition of nutrients and may lead to soil eutrophication. The proximity to settlement areas is greater for *Q. robur* forests, and such landscape features favor neophyte spread [19].

### 3.2. Importance of Soil Disturbance and Moisture Conditions

An additional factor contributing to the spread of neophytes was physical soil disturbance, such as soil scraping or compaction, caused by forest management interventions and natural disturbances. Soil disturbance had a positive effect on the plot-level species richness and cover of neophytes. Mechanical soil damage could provide opportunities for non-native invasive species to spread in forested landscapes by creating trails, spreading propagules and making available potentially new resources or niche spaces [38]. Šebesta et al. [20] reported that invasive plants were more abundant in sites impacted by mechanical soil disturbance. The importance of soil disturbance was emphasized particularly in microsites with increased exposure of mineral soil due to topsoil removal.

Management operations (tree felling, skidding, road construction) cause significant damage to trees and ground-surface layers, including ground vegetation and the upper-soil layer [39]. In our study, soil disturbance was mainly caused by the salvage logging of forest stands affected by tree mortality. The use of skidding trails and heavy forestry machinery greatly impacted the physical properties of the soil and at the same time acted as dispersal vectors for plant diaspores, i.e., seeds or vegetative parts [40]. On some plots, the soil was further disturbed by tree uprooting (windstorm damage) or local soil scraping caused by wild animals (e.g., wild boar). Such effects were identified as important drivers for the spread of neophytes in deciduous forests [37,41].

Soil conditions play an important role in explaining plant invasion patterns [42]. For instance, the water table level, in conjunction with tree canopy cover, was a decisive variable in the work of Lanta et al. [32]. Changes in soil conditions are not only relevant for the spread of neophytes but can also threaten the long-term viability of lowland *Quercus robur* forest ecosystems, particularly in relation to the natural hydrological regime and associated soil moisture availability. Across our study plots, oak mortality was the highest on moist sites where the groundwater level had significantly decreased [23]. On more mesic sites, intense forest management might favor the rejuvenation of the European hornbeam (*Carpinus betulus* L.), which often exhibits greater competitive ability and ecological plasticity compared to light-demanding oak seedlings/saplings. On wetter sites, however, large-scale disturbances and habitat degradation can lead to the conversion of oak forests to secondary forest associations dominated by black alder (*Alnus glutinosa*) in the tree layer and *Carex brizoides* L. in the herb layer. The removal of oak trees results in wetter soil conditions or even local waterlogging, conditions more favorable for black alder regeneration [43].

### 3.3. Impatiens parviflora and Other Frequent Neophytes

*Impatiens parviflora* was the most frequent neophyte and *Solidago gigantea* was the most abundant (i.e., a combination of frequency and average cover) across all resurveyed plots. The highest average percentage cover was recorded for *Erigeron annuus.* Their spread is assumed to be associated with the long-distance seed dispersal and exploitation of available resources in disturbed, open-canopy forests [32]. These widespread neophytes are considered highly invasive species in Slovenia and can be found in various forest types and non-forest habitats [44,45]. For example, ruderals such as *E. annuus* can successfully colonize canopy gaps in montane fir–beech forests [46], and *S. gigantea* is capable of forming dense clonal stands in a range of habitats.

**Figure 7:**
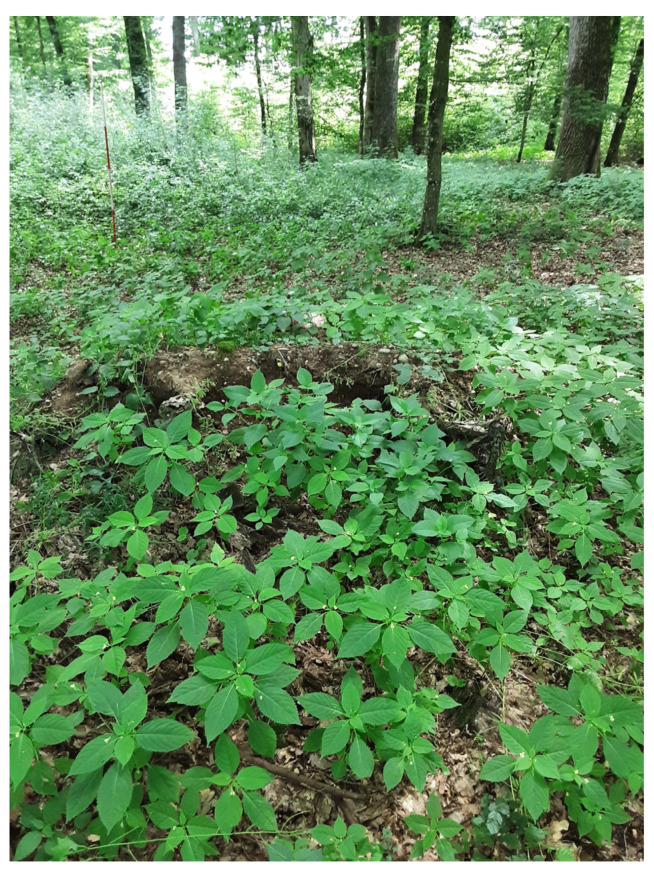
The spread of *Impatiens parviflora* in a relatively closed forest stand with some soil disturbance in a lowland oak–hornbeam forest dominated by *Quercus robur*, at the “Hraščica” study site. Photo: Janez Kermavnar.

We found that the number and abundance of neophytes decreased significantly with an increase in tree canopy cover. On some plots, however, the pattern of neophyte cover was skewed due to the presence of *I. parviflora*, a species capable of establishing in less disturbed stands under preserved canopy cover (Figure 7). *I. parviflora* occurred along the entire gradient of tree layer cover (serving as a proxy for light conditions in the understory). Whereas other neophytes established exclusively in disturbed patches with low tree layer cover and more available light, this neophyte was able to colonize relatively closed canopy stands on eutrophic and mesotrophic soils, where disturbance was minimal or absent.

*I. parviflora* is the most common neophyte species in all European forests [47]. It proliferates frequently in lowland deciduous forests [30,48,49] and other vegetation types, such as heathlands or scrub vegetation [50]. Its spread has been reported in several resurvey studies [5,41,51] and is known to negatively impact native vegetation in oak–hornbeam forests [48]. According to Chmura and Sierka [37], the penetration and establishment of this species in woodlands are facilitated by propagule pressure from habitats bordering forests (such as forest roads and edges), irradiance, gaps (empty space) in the herb layer, the presence of woody debris and rooting by wild boars. Additional factors could be atmospheric nitrogen deposition [52] or overstory shading effects [5]. This common neophyte employs an acquisitive resource-use strategy and has a high colonization capacity. Among the herbaceous flora of temperate forests, *I. parviflora* has an extremely high specific leaf area (i.e., over 140 mm^2^/mg according to the LEDA database; [53]). Such functional traits enable this species to establish in low-light conditions due to shade tolerance, thus avoiding competition.

Besides the aforementioned widespread neophytes, our resurvey study revealed the potential threat of some other neophytes not yet well recognized. An example of this is *Erechtites hieraciifolia*, a non-native species from North America. *E. hieraciifolia* (American fireweed) is an annual herb that usually behaves as a pioneer species, colonizing canopy openings in coniferous and deciduous forests. In Slovenia, the distribution of this species is not sufficiently known; it is probably underestimated and has been overlooked due to its relatively late flowering and similarity to native ragworts (*Senecio* spp.). In recent decades, its occurrence has evidently been increasing in disturbed forests in Central and Eastern Europe [54]. This neophyte was frequently present in disturbed stands affected by wildfire in the Sub-Mediterranean phytogeographic region of Slovenia in 2022 [55]. Its occurrence in moist lowland oak–hornbeam forests (this study) and on dry skeletal soils on karst terrain suggest its broad ecological amplitude, a characteristic common to many highly proliferating neophytes. Gaining occurrences of certain species may call for an update of official, nationwide warning lists for invasive plants developed within specific projects (e.g., [44,45]).

The rapid colonization of canopy gaps by herbaceous neophytes can also be explained by their functional traits [56], as the most abundant neophytes were competitive ruderals with high dispersal capacity and an acquisitive resource-use strategy. Among native herbaceous species, perennials with primarily vegetative reproduction for local persistence prevail, whereas many neophytes are annuals. Furthermore, the flowering period of neophytes is considerably longer, and seed production is much higher [56]. However, there are exceptions to this rule because some proliferating neophytes show a high degree of plasticity and can invade closed-canopy forest stands.

Although the understories of intensively disturbed *Q. robur* plots were highly dominated by dense stands of native graminoids (e.g., *Carex brizoides*, *Deschampsia cespitosa* (L.) P. Beauv., *Juncus effusus* L., *Agrostis canina* L.), neophytes were still able to colonize newly created canopy openings. This suggests that abiotic factors were more important as determinants of invasion success than biotic factors (competition induced by denser native vegetation in the herb layer). However, the most invaded sites in lowland *Q. robur* forests were generally species-poor communities, which might suggest that assemblages with a higher number of resident species may be less prone to invasion (e.g., [32]).

## 4. Materials and Methods

### 4.1. Study Area

In the early 1990s, a network of permanent research plots in managed, semi-natural oak forests was established [57]. Five sites in lowland oak–hornbeam forests dominated by *Quercus robur* and four sites in colline oak–beech forests dominated by *Quercus petraea* were selected. The majority of sites are located in the Sub-Pannonian phytogeographic region of Slovenia. All nine sites were characterized by late-successional forest complexes with a stand age of at least 80 years, closed tree layer canopy (95% or above) and homogenous ecological conditions.

Lowland oak–hornbeam forests are located on flat floodplains of rivers where deep hydromorphic soils prevail. These forest sites are naturally periodically flooded and under the constant influence of high groundwater levels, or at least saturated with water for some time period [43]. The mean annual temperature across the five sites in the period 1961–1990 was 9.9 °C, and the mean annual precipitation was 1023 mm. Based on the field inventory in 1992/93, the stand density (the number of trees with DBH ≥ 10 cm) ranged between 305 and 623 trees per hectare. The stand growing stock ranged between 300 and 680 m^3^/ha. The proportion of *Quercus robur* in the growing stock was 79.8% on average. Admixed species in the tree layer included *Carpinus betulus*, *Picea abies* (L.) Karst., *Tilia cordata* Mill., *Alnus glutinosa* and *Acer campestre* L. [57,58,59,60].

The selected colline oak–beech forests are situated on permeable hilly terrain with undulating topography. Different types of soil are formed on limestone, flysch or sandy clay parent material. The mean annual temperature across the four sites in the period 1961–1990 was 10.3 °C, and the mean annual precipitation was 1199 mm. The stand density ranged between 305 and 648 trees per hectare. Based on the field inventory in 1992/93, the stand growing stock ranged between 307 and 589 m^3^/ha. The proportion of *Quercus petraea* in the growing stock was 88.6% on average. Frequently admixed tree species in the overstory included *Fagus sylvatica* L., *Carpinus betulus*, *Quercus cerris* L. and *Prunus avium* L. [57,58,59,60]. The general characteristics of the selected sites are presented in Table 4.

### 4.2. Sampling Design and Vegetation Survey

**Figure 8:**
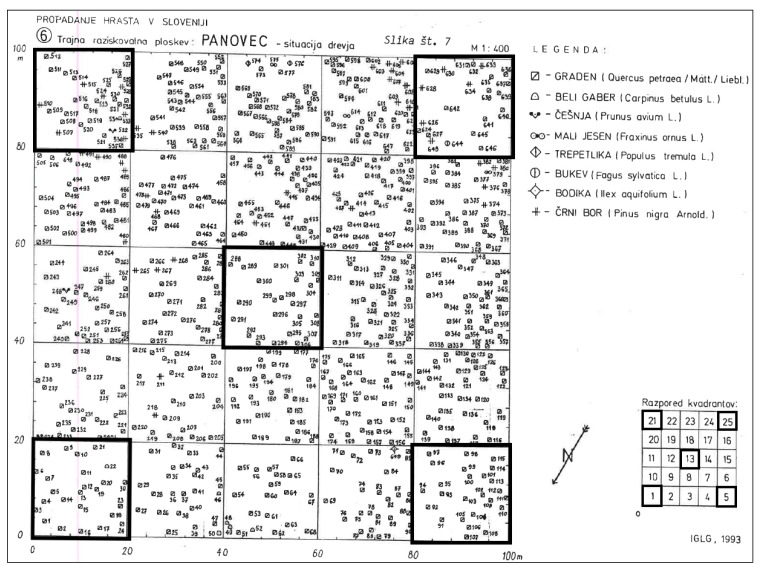
A detailed map of the spatial distribution of numbered trees across sampling plots in the “Panovec” 1 ha research area in the colline oak–beech forest dominated by *Quercus petraea.* This reflects the situation in the original survey in 1992 (adapted from [58]). The five bolded 20 × 20 m plots were resurveyed in 2023.

At each site, a one-hectare (100 × 100 m) permanent research area with well-preserved, homogenous stand conditions was established in the central part of the forest complex [57,58]. The research area was divided into 25 sampling plots, each measuring 20 × 20 m. In the 1992/93 survey, all trees with DBH ≥ 10 cm were measured, numbered and mapped, providing a detailed spatial scheme of the stand situation (Figure 8).

In the summer of 2023, the permanent plots in oak–hornbeam (dominated by *Q. robur*) and oak–beech (dominated by *Q. petraea*) forests were revisited. Field sampling followed the original survey methods [58] in precisely located permanent plots with no relocation error. The reconstruction of more disturbed plots was still possible based on existing marked trees and tree stumps. At each site, we collected the vegetation data on five plots, i.e., the central plot and four plots in corners (bolded quadrats in Figure 8). This resulted in 25 plots across *Q. robur* and 20 plots across *Q. petraea* forests, totaling 45 pairs of old and new relevés.

Complete floristic surveys (phytosociological relevés) of all vascular plants in the understory layer were conducted in 1992/93. Understory vegetation in this study was defined as all herbaceous species and woody plants in the herb and shrub layer (height less than 5 m). The understory vegetation of the research plots was surveyed according to the standard Central European phytosociological method. The percentage cover of each vascular plant species in the shrub and herb layer was estimated using the Braun-Blanquet seven-degree cover class scale [61]. In addition, the tree layer canopy cover (all woody species taller than 5 m), as a measure of canopy closure, was estimated visually on each plot. Vegetation resurveys were carried out at the peak of understory development and following the exact same protocol as in the original surveys [58] to ensure a high degree of comparability. The source for the species nomenclature was the National Flora [62].

### 4.3. Definition of Neophytes

Species introduced intentionally or unintentionally by humans to new biogeographical regions from other continents are classified as non-native (alien) plants. In this study, neophytes are defined as non-native plant species introduced to Europe or its sub-regions after 1500 AD. For each recorded species in the understory layer across our plots, the status in Europe was assessed according to the origin of the species using the FloraVeg.EU database (Database of European Vegetation, Habitats and Flora; [63]), distinguishing between native species and neophytes, regardless of their position along the introduction–naturalization–invasion continuum (as per [64]).

### 4.4. Data Analysis

This study is focused on the occurrence of neophytes in permanent vegetation plots in two oak-dominated forest types. For the 1992/93 survey and 2023 resurvey, we calculated the total number of recorded neophytes across all plots in lowland oak–hornbeam forests dominated by *Quercus robur* and in colline oak–beech forests dominated by *Quercus petraea*. The two main response variables were the plot-level richness (the number of neophytes in a plot) and plot-level cover of neophytes (in %). The latter variable represents the cumulative cover of all neophytes in a plot, obtained by summing the abundance percentages. For this analysis, abundance data estimated by the Braun-Blanquet cover class scale [61] were converted to respective mid-point values as follows: r = 0.1%; + = 0.5%; 1 = 3%; 2 = 15%; 3 = 37.5%; 4 = 62.5%; and 5 = 87.5%. The statistical significance of differences between the 1992/93 survey and 2023 resurvey was tested using the Wilcoxon signed-rank test for paired data [65].

To explore how oak mortality influenced the occurrence of neophytes, their plot-level richness and abundance were correlated with tree layer canopy cover (estimated during the field resurvey). In addition, a vegetation-derived estimate of soil disturbance was calculated for each plot. Disturbance indicator values were sourced from [66], and community-weighted means (CWMs; [67]) were computed based on the understory composition of all recorded species (natives and neophytes) in the 2023 resurvey. For a continuous trait, the CWM is the mean trait value of all understory species present in the community (plot), weighted by their relative abundances. The “functcomp” function in the FD package [68] was used for this purpose.

The relationship between the plot-level richness of neophytes and two explanatory variables (tree layer cover, soil disturbance) was quantified using a generalized linear model. Since the dependent variable represents count data, a quasi-Poisson error distribution (due to overdispersion) and logarithmic link function were used in model specifications (“glm” function in stats package). The response of the plot-level cover of neophytes along the tree layer cover and soil disturbance gradient was assessed by constructing a generalized linear model with a quasi-binomial distribution family. All models were checked for heteroskedasticity, normality in raw residuals and zero-inflation.

We investigated the response of the most frequent neophytes to selected environmental factors (predictors) with species response curves, given that individual species are likely to experience non-linear distributions along environmental gradients. We used the “specresponse” function implemented in the goeveg R package [69] to generate response curves. In this analysis for the data subset including plots from *Quercus robur* forests, predictors included tree layer cover (%), CWMs for disturbance severity, disturbance frequency and soil disturbance and the relative change in CWMs of ecological indicator values for light, temperature, soil moisture and soil nutrients (nitrogen). Disturbance severity and disturbance frequency values were calculated as CWMs in the same way as for soil disturbance. Disturbance indicator values for plants, expressing species positioning along natural and anthropogenic disturbance gradients, were sourced from Midolo et al. [66]. Ecological indicator values of plant species were taken from the EIVE 1.0 database [70]. The phytoindication-based approach, widely used to indirectly estimate abiotic site conditions [71], has frequently been utilized to explore the occurrence patterns of neophytes along environmental gradients (e.g., [30,49]). The relative change for each ecological factor (light, temperature, soil moisture and soil nutrients/nitrogen) was calculated as the percentage change in the CWM between the 1992/93 survey and the 2023 resurvey, with positive relative change indicating an increase in resource availability over time and a negative relative change suggesting a decrease. The “functcomp” function in the FD package was used for computing CWM values based on all understory species recorded in a plot.

All analyses and data visualizations were performed in R software version 4.3.0 [72].

## 5. Conclusions

We studied long-term changes in semi-natural oak forests in Slovenia by resampling 45 permanent vegetation plots that were first surveyed in the early 1990s. The richness and abundance of neophytes increased over the last 30 years, with their spontaneous colonization mainly triggered by habitat degradation. The highest level of neophyte proliferation was observed in plots with an extensive loss of tree layer cover, accompanied by wind damage and salvage logging that caused soil disturbance. Lowland *Quercus robur* forests exhibited a significantly higher degree of neophyte proliferation compared to colline *Quercus petraea* forests. The main contributing factor was the higher mortality rate of pedunculate oak and overall greater disturbance effects in the former forest type.

Due to climate change and forest disturbances, the spread of neophytes is expected to intensify in the future in Slovenian oak forests as well as in other forest types. The rapid spread of neophytes may be perceived as one of the symptoms of anthropogenic global change, threatening the ecological integrity, ecosystem sustainability and long-term perspective [12] of oak forests in lowland areas [22]. The recorded neophytes are mostly early successional species, and hence many of them are expected to be excluded during the first 10–20 years of uninterrupted secondary succession. However, *Impatiens parviflora* was able to colonize less disturbed patches due to its shade tolerance, indicating that not all neophytes require a high-light environment for establishment. Such species pose a particular management challenge, as they can spread even in non-degraded forest stands. Nevertheless, preserving tree layer cover with sufficient microclimatic buffering and minimizing soil disturbance are key factors that can contribute to resistance against the spread of neophytes [9].

The further monitoring and control of the spread and impacts of neophytes are necessary. Long-term resurvey studies in forests experiencing an overall degradation of habitat conditions should focus on temporal trends in neophytes, preferably using permanent vegetation plots in different forest vegetation types.

## Figures and Tables

**Table 1:** Neophytes in lowland oak–hornbeam forests dominated by *Quercus robur*. Presence—the number of plots on which a species was recorded. Mean cover—the averaged cover (%) on plots where the species occurred. Species are ordered according to their occurrence and then by mean cover. Species with the same occurrence frequency and mean cover are listed alphabetically.

Species	Survey 1992/93		Resurvey 2023	
	Presence	Mean Cover (%)	Presence	Mean Cover (%)
*Impatiens parviflora*	0	0	14	5.1
*Solidago gigantea*	0	0	12	7.2
*Erechtites hieraciifolia*	0	0	7	1.6
*Erigeron annuus*	0	0	6	7.5
*Impatiens glandulifera*	0	0	4	4.8
*Epilobium ciliatum*	0	0	4	1.8
*Veronica persica*	3	0.5	1	0.5
*Conyza canadensis*	0	0	3	1.4
*Bidens frondosa*	0	0	3	0.5
*Phytollaca americana*	0	0	2	0.5
*Oxalis fontana*	0	0	2	0.5
*Pinus strobus*	1	0.5	1	0.5
*Duchesnea indica*	0	0	1	0.5
*Juglans nigra*	0	0	1	0.5
*Matricaria discoidea*	0	0	1	0.5
*Parthenocissus quinquefolia*	0	0	1	0.5
*Populus* × *canadensis*	0	0	1	0.5
*Robinia pseudacacia*	0	0	1	0.5
*Solidago canadensis*	0	0	1	0.5
*Quercus rubra*	0	0	1	0.5

**Table 2:** Neophytes in colline oak–beech forests dominated by *Quercus petraea*. Presence—the number of plots on which a species was recorded. Mean cover—the averaged cover (%) on plots where the species occurred.

Species	Survey 1992/93		Resurvey 2023	
	Presence	Mean Cover (%)	Presence	Mean Cover (%)
*Conyza canadensis*	1	0.5	1	0.5
*Erigeron annuus*	0	0	1	0.5
*Galinsoga parviflora*	0	0	1	0.5
*Robinia pseudacacia*	0	0	1	0.5
*Solidago canadensis*	0	0	1	0.5
*Solidago gigantea*	0	0	1	0.5
*Spiraea japonica*	0	0	1	0.5

**Table 3:** The percentage of deviance explained for species response curves for the two most frequent neophytes in lowland oak–hornbeam forests dominated by *Quercus robur*, i.e., *Impatiens parviflora* and *Solidago gigantea*. Significance levels are coded as follows: * *p* < 0.05, ** *p* < 0.01, *** *p* < 0.001, ns—non-significant. Refer to Figure 5 for response curves.

Ecological Factor	*Impatiens parviflora*	*Solidago gigantea*
Tree layer cover	41.9 **	52.5 ***
Disturbance severity	50.7 ***	61.7 ***
Disturbance frequency	53.9 ***	64.3 ***
Soil disturbance	56.2 ***	51.5 ***
Light—relative change	40.5 ***	68.0 ***
Temperature—relative change	2.9 ^ns^	1.5 ^ns^
Moisture—relative change	21.5 *	49.1 ***
Nutrients—relative change	8.2 ^ns^	5.5 ^ns^

**Table 4:** General characterization of oak study sites in Slovenia. Information was summarized from previous reports and publications [57,58,59,60].

Site	Latitude, Longitude	Elevation (m a.s.l.)	Dominant Tree Species (% in Growing Stock 1990s)	Study Period
1. Krakovski gozd	45.8819, 15.4159	150	*Quercus robur* (70.5%)	1992–2023
2. Cigonca	46.3633, 15.5816	260	*Quercus robur* (80.1%)	1992–2023
3. Hraščica	46.6439, 16.2780	180	*Quercus robur* (83.7%)	1992–2023
4. Dobrava	45.9426, 15.6520	160	*Quercus robur* (87.2%)	1993–2023
5. Polom	45.7483, 14.8616	370	*Quercus robur* (77.5%)	1992–2023
6. Bojanci	45.4938, 15.2671	280	*Quercus petraea* (98.4%)	1992–2023
7. Panovec	45.9492, 13.6750	140	*Quercus petraea* (95.4%)	1992–2023
8. Bukovnica	46.6996, 16.3251	230	*Quercus petraea* (82.3%)	1993–2023
9. Pišece	46.0128, 15.6538	470	*Quercus petraea* (78.1%)	1993–2023

## Data Availability

The datasets generated during and/or analyzed during the current study are available from the corresponding author upon reasonable request.
